# A New Insight into Meloxicam: Assessment of Antioxidant and Anti-Glycating Activity in In Vitro Studies

**DOI:** 10.3390/ph13090240

**Published:** 2020-09-10

**Authors:** Cezary Pawlukianiec, Małgorzata Ewa Gryciuk, Kacper Maksymilian Mil, Małgorzata Żendzian-Piotrowska, Anna Zalewska, Mateusz Maciejczyk

**Affiliations:** 1 Students Scientific Club “Biochemistry of Civilization Diseases” at the Department of Hygiene, Epidemiology and Ergonomics, Medical University of Bialystok, 2c Mickiewicza Street, 15-233 Bialystok, Poland; cezary.pawlukianiec@gmail.com (C.P.); mgryciuk1@gmail.com (M.E.G.); mil.kacper@wp.pl (K.M.M.); 2 Department of Hygiene, Epidemiology and Ergonomics, Medical University of Bialystok, 2c Mickiewicza Street, 15-233 Bialystok, Poland; mzpiotrowska@gmail.com; 3 Experimental Dentistry Laboratory, Medical University of Bialystok, 24a M. Sklodowskiej-Curie Street, 15-274 Bialystok, Poland; azalewska426@gmail.com

**Keywords:** meloxicam, protein glycation, protein oxidation, protein glyco-oxidation, antioxidant activity

## Abstract

Meloxicam is a non-steroidal anti-inflammatory drug, which has a preferential inhibitory effect to cyclooxyganase-2 (COX-2). Although the drug inhibits prostaglandin synthesis, the exact mechanism of meloxicam is still unknown. This is the first study to assess the effect of meloxicam on protein glyco-oxidation as well as antioxidant activity. For this purpose, we used an in vitro model of oxidized bovine serum albumin (BSA). Glucose, fructose, ribose, glyoxal and methylglyoxal were used as glycating agents, while chloramine T was used as an oxidant. We evaluated the antioxidant properties of albumin (2,2-di-phenyl-1-picrylhydrazyl radical scavenging capacity, total antioxidant capacity and ferric reducing antioxidant power), the intensity of protein glycation (Amadori products, advanced glycation end products) and glyco-oxidation (dityrosine, kynurenine, N-formylkynurenine, tryptophan and amyloid-β) as well as the content of protein oxidation products (advanced oxidation protein products, carbonyl groups and thiol groups). We have demonstrated that meloxicam enhances the antioxidant properties of albumin and prevents the protein oxidation and glycation under the influence of various factors such as sugars, aldehydes and oxidants. Importantly, the antioxidant and anti-glycating activity is similar to that of routinely used antioxidants such as captopril, Trolox, reduced glutathione and lipoic acid as well as protein glycation inhibitors (aminoguanidine). Pleiotropic action of meloxicam may increase the effectiveness of anti-inflammatory treatment in diseases with oxidative stress etiology.

## 1. Introduction

An integral part of cellular oxygen metabolism is the production of reactive oxygen species (ROS) [1]. In low concentrations, oxygen free radicals are involved in many physiological processes; nevertheless, under long-term exposure to pro-oxidant agents, the formation of ROS becomes greater than the ability of cells to eliminate them [1]. Disturbances in oxidative-reductive homeostasis (in favor of the oxidation reactions) result in oxidative stress. This process leads to oxidative damage to biomolecules such as proteins, lipids and DNA and is believed to be the cause or effect of several contemporary diseases [2,3,4]. The low probability of direct ROS reactions with lipids and DNA indicates that proteins are the most important target for free radicals. Indeed, in a typical mammalian cell, over 70% of hydroxyl radicals (•OH) react with proteins [5].

Critical processes that change the metabolism and organization of cells are oxidation and glycation of proteins [5]. Free radicals are responsible for the oxidation of single amino acid residues or a polypeptide chain, which can lead to its breaking up as well as cross-linking within the same or more polypeptide chains [5,6]. It has been shown that oxidized proteins can accumulate and aggregate in tissues as well as lose their biological activity [5,6]. Glycation is a non-enzymatic multistage reaction between reducing sugars and proteins [7]. This process is usually catalyzed by pro-oxidant metals and is closely related to ROS production and oxidation. Therefore, glycation and oxidation are collectively referred to as glyco-oxidation [3,8]. At the beginning, the carbonyl group of carbohydrate reacts with free amino group of proteins giving the Schiff base as a product. The next step is rearranging Schiff base into ketamine or Amadori products that, after reactions such as dehydration, cyclization, and oxidation, form advanced glycation end products (AGEs) [6,9]. The glycation process takes place in every cell of the body; nevertheless, in many pathological processes (especially during hyperglycemia) glycation increases [8]. Although the share of glycated albumin in healthy people varies between 1% and 10%, it may even increase several times in diabetes and other metabolic diseases [10]. It is therefore not surprising that glycated albumin is used as a short- and medium-term glycemic control marker [10]. Oxidized albumin is also used in many in vitro studies as a model of glyco-oxidation [8,9,11,12]. Under these conditions, there is an increased formation of AGEs, cross-linking of aromatic amino acids and changes in their fluorescence.

It is proven that both ROS and advanced glycation end products are capable of altering cells’ structure and functioning which often leads to damage of cellular DNA, excessive cell proliferation, genomic instability, as well as deactivation of enzymes [13,14]. Interestingly, excessive glycation and oxidation of proteins are important factors in the pathogenesis of aging as well as metabolic, autoimmune, neurodegenerative and inflammatory diseases [15,16,17,18,19]. Therefore, it is not surprising that substances with antioxidant and anti-glyco-oxidant properties are still being sought. Of particular interest are molecules that are already widely used. Indeed, their safety profile and pharmacokinetic/pharmacodynamic characteristics are well known and thus they can be used for additional indications.

**Figure 1:**
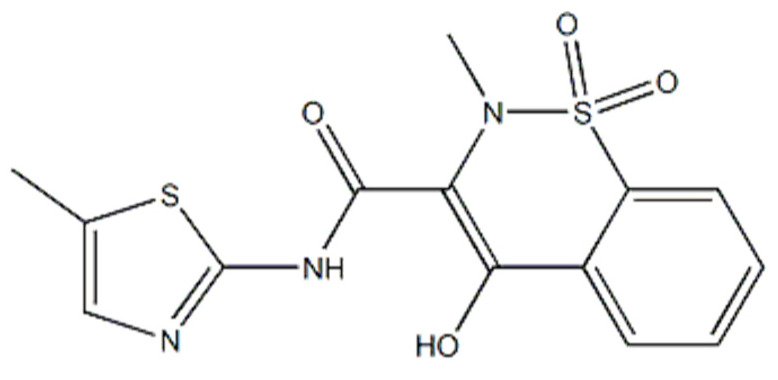
Chemical structure of meloxicam.

The results of recent studies indicate the potential antioxidant properties of meloxicam. Meloxicam (4-hydroxy-2-methyl-N-(5-methyl-1,3-thiazol-2-yl)-1,1-dioxo-1^λ^6,2-benzothiazine-3-carboxamide, C_14_H_13_N_3_O_4_S_2_, Figure 1) is one of the non-steroidal anti-inflammatory drugs (NSAIDs), often used in inflammatory diseases such as rheumatoid arthritis or osteoarthritis [20]. Meloxicam is a preferential inhibitor of cyclooxygenase-2 (COX-2). Indeed, by inhibiting COX-2, meloxicam prevents the conversion of arachidonic acid into pro-inflammatory prostaglandins [21]. However, this drug may also show other biological activity. For example, it is reported that meloxicam protects from hepatotoxicity [22] or inhibits cell apoptosis by strengthening the antioxidant barrier [23]. What is more, the same mechanism was said to be responsible for neuroprotective actions of the drug [24]. However, despite some reports of potential antioxidant effects of meloxicam, it is still unclear whether this drug can prevent protein glycation and oxidation. Therefore, the aim of the study was to assess the effect of meloxicam on protein glyco-oxidation as well as antioxidant activity. For this purpose, we used an in vitro model of oxidized bovine serum albumin (BSA). On the basis of literature analysis, we have selected the most frequently used glycating (glucose, fructose, ribose, glyoxal and methylglyoxal) and oxidizing agents (chloramine T) [8,9,11,12,25,26,27,28]. We compared the effectiveness of meloxicam with recognized free radical scavengers (captopril, Trolox, reduced glutathione and lipoic acid) as well as protein glycation inhibitors (aminoguanidine and metformin) [8,9,27,28,29]. We evaluated the antioxidant properties of albumin (DPPH, TAC, FRAP), the intensity of protein glycation (Amadori products, AGE) and glyco-oxidation (dityrosine, kynurenine, N-formylkynurenine, tryptophan and amyloid) as well as the content of protein oxidation products (AOPP, PC and thiol groups).

## 2. Results

### 2.1. The Effects of Meloxicam and Other Additives on Protein Glycation, Glycooxidation and Oxidative Damage as Well as Total Antioxidant Potential in Glucose (Glc)-Induced Albumin Glycation

**Figure 2:**
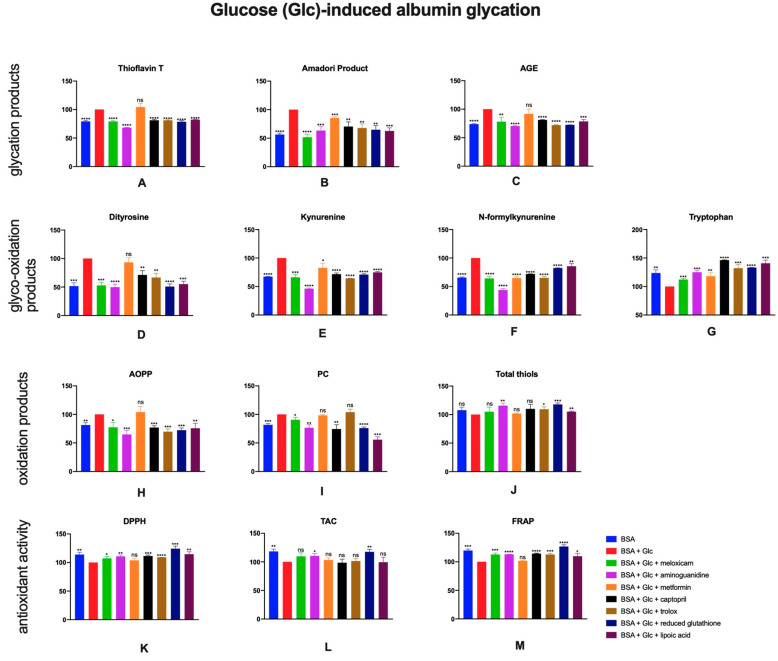
The effects of meloxicam, captopril, trolox, reduced glutathione, lipoic acid, aminoguanidine or metformin addition to BSA + glucose solution on protein glycation (**A**–**C**), glycooxidation (**D**–**G**) and oxidative damage (**H**–**J**) as well as total antioxidant potential (**K**–**M**). AGE: advanced glycation end products; AOPP: advanced oxidation protein products; BSA: bovine serum albumin; DPPH: 2,2-di-phenyl-1-picrylhydrazyl radical scavenging capacity; FRAP: ferric reducing antioxidant power; Glc: glucose; PC: protein carbonyls; TAC: total antioxidant capacity; ns: not significant vs. control; * *p* < 0.05 vs. control; ** *p* < 0.01 vs. control; *** *p* < 0.001 vs. control; **** *p* < 0.0001 vs. control.

Glucose-induced glycation caused an increase in levels of products of these processes—thioflavin T, Amadori products, AGE. Addition of aminoguanidine (↓32%, ↓37%, ↓30%, respectively) captopril (↓19%, ↓30%, 19%), trolox (↓19%, ↓32%, ↓28%), reduced glutathione (↓21%, ↓35%, ↓28%) or lipoic acid (↓18%, ↓37%, ↓21%) and also meloxicam (↓21%, ↓48%, ↓22%) resulted in lower concentrations of glycation products (Figure 2A–C).

The presence of glucose resulted in increased contents of protein glyco-oxidation products—dityrosine, kynurenine, N-formylkynurenine. When glyco-oxidation or glycation inhibitors were added (aminoguanidine, captopril, trolox, reduced glutathione or lipoic acid), a decrease of levels of the aforementioned products were observed. Meloxicam also presented anti-glyco-oxidative properties as the content of products of this process was significantly lower in samples containing the drug (↓47%, ↓34%, ↓36%, respectively). However, concentration of tryptophan was significantly higher compared to the control sample (Figure 2D–G).

Regarding oxidation products, the addition of glucose to BSA resulted in higher AOPP and PC levels. Investigated inhibitors, along with meloxicam (↓23%, ↓10%, respectively), caused a decrease of these parameters. Total thiols change in sample containing meloxicam was insignificant (Figure 2H–J).

Addition of meloxicam to BSA + Glc resulted in increased DPPH and FRAP comparing to control. Significantly higher TAC was observed also in BSA, BSA + Glc with aminoguanidine or reduced glutathione in comparison to control (Figure 2K–M).

### 2.2. The Effects of Meloxicam and Other Additives on Protein Glycation, Glycooxidation and Oxidative Damage as Well as Total Antioxidant Potential in Fructose (Fru)-Induced Albumin Glycation

**Figure 3:**
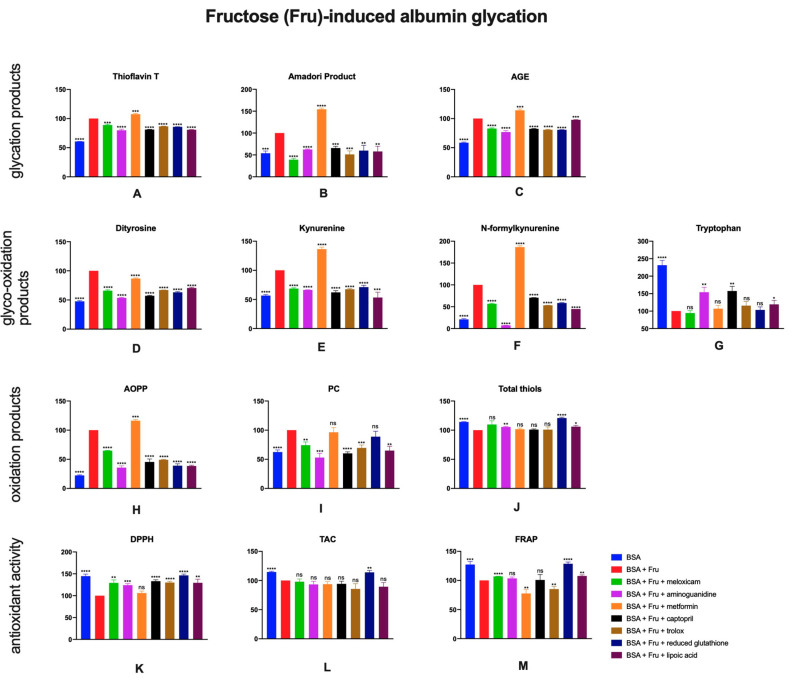
The effects of meloxicam, captopril, trolox, reduced glutathione, lipoic acid, aminoguanidine or metformin addition to BSA + fructose solution on protein glycation (**A**–**C**), glycooxidation (**D**–**G**) and oxidative damage (**H**–**J**) as well as total antioxidant potential (**K**–**M**). AGE: advanced glycation end products; AOPP: advanced oxidation protein products; BSA: bovine serum albumin; DPPH: 2,2-di-phenyl-1-picrylhydrazyl radical scavenging capacity; FRAP: ferric reducing antioxidant power; Fru: fructose; PC: protein carbonyls; TAC: total antioxidant capacity; ns: not significant vs. control; * *p* < 0.05 vs. control; ** *p* < 0.01 vs. control; *** *p* < 0.001 vs. control; **** *p* < 0.0001 vs. control.

Protein glycation products were present at higher concentrations where fructose was used as a glycating agent. Aminoguanidine (↓20%, ↓38%, ↓23%, respectively), captopril (↓19%, ↓34%, ↓18%), trolox (↓13%, ↓49%, ↓19%) or lipoic acid (↓19%, ↓42%, ↓2%) and also meloxicam (↓11%, ↓61%, ↓17%) caused a decrease in thioflavin T, Amadori products and AGE levels compared to control (Figure 3A–C).

Fructose-induced albumin glyco-oxidation, similarly to glucose, effected in significantly higher concentrations of glyco-oxidation products—dityrosine, kynurenine and N-formylkynurenine. Presence of aminoguanidine (↓46%, ↓34%, ↓92,9%, respectively), captopril (↓43%, ↓38%, 29%), trolox (↓33%, ↓32%, ↓47%), reduced glutathione (↓37%, ↓29%, ↓41%) or lipoic acid (↓29%, ↓47%, ↓55%) in BSA + Fru sample resulted in a decreased level of glyco-oxidation markers in comparison to the control. Action of meloxicam (↓34%, ↓31%, ↓43%) was similar to used inhibitors—the addition of the drug led to less protein damage measured by the parameters above. In contrast, samples with metformin showed higher contents of kynurenine, N-formylkynurenine and thioflavin T (↑36%, ↑87%, ↑8%, respectively) versus BSA + Fru (Figure 3D–G).

Parameters of oxidative damage (AOPP and PC) were greater in presence of fructose compared to BSA alone. All added substances (excluding metformin) caused a significant decrease in levels of oxidation products (Figure 3H–J).

Regarding total antioxidant potential assays, BSA + Fru + meloxicam sample was characterized by significantly greater DPPH and FRAP parameters (↑29%, ↑7%, respectively), compared to control sample. TAC was significantly higher also in BSA alone and BSA + Fru + reduced glutathione (↑14%) (Figure 3K–M).

### 2.3. The Effects of Meloxicam and Other Additives on Protein Glycation, Glycooxidation and Oxidative Damage as Well as Total Antioxidant Potential in Ribose (Rib)-Induced Albumin Glycation

**Figure 4:**
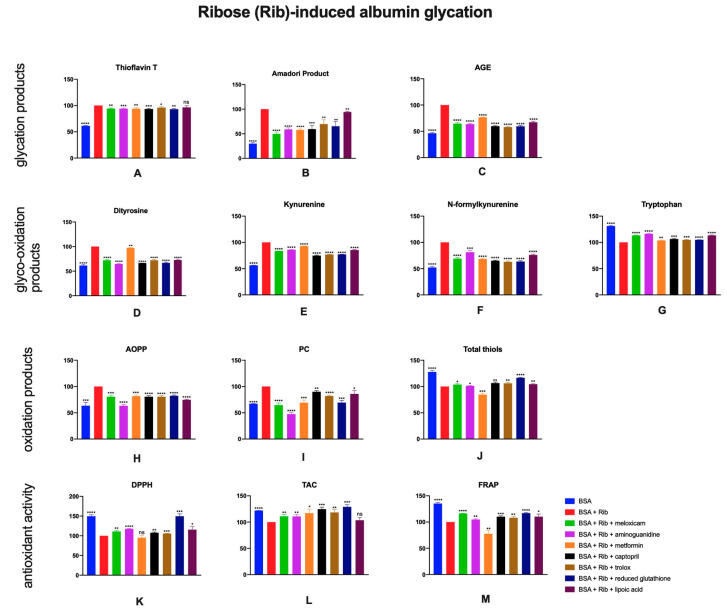
The effects of meloxicam, captopril, trolox, reduced glutathione, lipoic acid, aminoguanidine or metformin addition to BSA + ribose solution on protein glycation (**A**–**C**), glycooxidation (**D**–**G**) and oxidative damage (**H**–**J**) as well as total antioxidant potential (**K**–**M**). AGE: advanced glycation end products; AOPP: advanced oxidation protein products; BSA: bovine serum albumin; DPPH: 2,2-di-phenyl-1-picrylhydrazyl radical scavenging capacity; FRAP: ferric reducing antioxidant power; PC: protein carbonyls; Rib: ribose; TAC: total antioxidant capacity; ns: not significant vs. control; * *p* < 0.05 vs. control; ** *p* < 0.01 vs. control; *** *p* < 0.001 vs. control; **** *p* < 0.0001 vs. control.

The presence of ribose in BSA solution resulted in higher glycation products content. The analysis showed significantly lower amounts of thioflavin T, Amadori products and AGE in BSA + Rib + meloxicam (↓6%, ↓50%, ↓33%, respectively), in comparison to the control sample without the investigated drug. Samples with remaining glycation inhibitors also presented decreased levels of Amadori products and AGE (Figure 4A–C).

Ribose-induced albumin glyco-oxidation resulted in higher contents of dityrosine, kynurenine and N-formylkynurenine. Lower levels of given substances were notice in BSA + Rib + aminoguanidine (↓35%, ↓14%, ↓19%, respectively), metformin (↓3%, ↓7%, ↓32%), captopril (↓33%, ↓25%, ↓35%), trolox (↓28%, ↓23%, ↓37%), reduced glutathione (↓33%, ↓323%, ↓36%), and lipoic acid (↓27%, ↓15%, ↓24%) samples. The anti-glyco-oxidative character of meloxicam was also proven, as the contents of glyco-oxidation damage products were significantly lower (↓28%, ↓16%, ↓31%) in samples containing the drug. In addition, content of tryptophan was increased in BSA + Rib + meloxicam (↑13%), aminoguanidine (↑16%), metformin (↑4%), captopril (↑6%), trolox (↑5%), reduced glutathione (↑5%) or lipoic acid (↑13%) comparing to control (Figure 4D–G).

Ribose-induced oxidation led to higher concentrations of AOPP and PC. Used inhibitors diminished this action and decreased given parameters. Total thiols concentrations were statistically higher compared to control, excluding BSA + Rib + metformin sample, where a decrease was observed (Figure 4H–J).

The BSA + Rib + meloxicam sample was characterized by significantly greater DPPH, TAC and FRAP (↑11%, ↑11%, ↑16%, respectively) parameters, compared to BSA + Rib sample. Moreover, all the samples with oxidation inhibitors had higher TAC compared to control. Similar results were obtained regarding FRAP (except metformin). Increased DPPH was observed in samples with aminoguanidine and reduced glutathione (↑18%, ↑50%, Figure 4K–M).

### 2.4. The Effects of Meloxicam and Other Additives on Protein Glycation, Glycooxidation and Oxidative Damage as Well as Total Antioxidant Potential in Glyoxal (GO)-Induced Albumin Glycation

**Figure 5:**
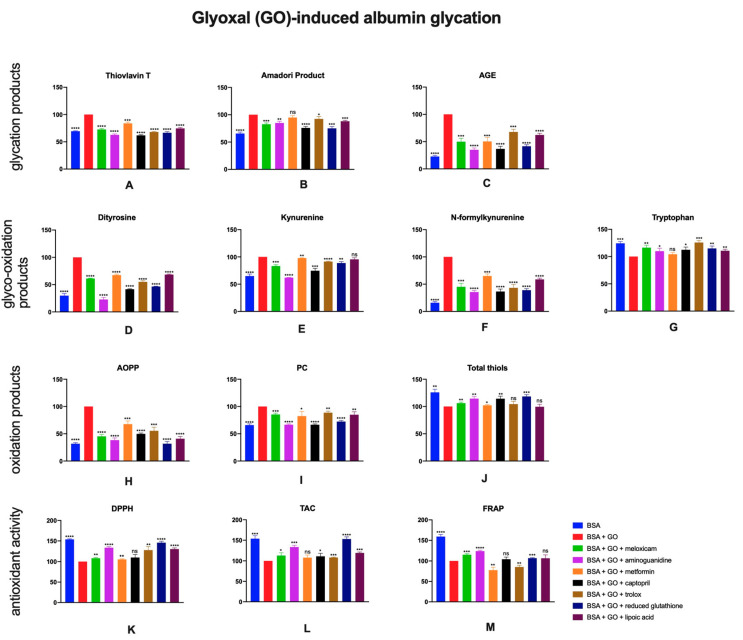
The effects of meloxicam, captopril, trolox, reduced glutathione, lipoic acid, aminoguanidine or metformin addition to BSA + glyoxal solution on protein glycation (**A**–**C**), glycooxidation (**D**–**G**) and oxidative damage (**H**–**J**) as well as total antioxidant potential (**K**–**M**). AGE: advanced glycation end products; AOPP: advanced oxidation protein products; BSA: bovine serum albumin; DPPH: 2,2-di-phenyl-1-picrylhydrazyl radical scavenging capacity; FRAP: ferric reducing antioxidant power; GO: glyoxal; PC: protein carbonyls; TAC: total antioxidant capacity; ns: not significant vs. control; * *p* < 0.05 vs. control; ** *p* < 0.01 vs. control; *** *p* < 0.001 vs. control; **** *p* < 0.0001 vs. control.

The presence of glyoxal in BSA effected in the increase of glycation markers. The analysis of glycation damage showed significantly lower contents of thioflavin T, Amadori products and AGE in BSA + GO + meloxicam (↓27%, ↓17%, ↓50%, respectively) comparing to BSA + GO. Moreover, in samples with addition of protein glycation and oxidation inhibitors, similar results, excluding Amadori products in sample with metformin, were observed. Significantly higher values of total thiols in solutions including meloxicam (↑6%), aminoguanidine (↑14%), metformin (↑2%), captopril (↑12%) or reduced glutathione (↑26%) were detected (Figure 5A–C).

Glyco-oxidation of albumin induced by glyoxal resulted in greater amounts of dityrosine, kynurenine and N-formylkynurenine. The addition of the anti-glyco-oxidative agents (aminoguanidine (↓77%, ↓38%, ↓64%), metformin (↓32%. ↓2%, ↓35%), captopril (↓58%, ↓25%, ↓63%), trolox (↓45%, ↓9%, ↓57%), reduced glutathione (↓53%, ↓11%, ↓61%), lipoic acid (↓32%, ↓4%, ↓41%)) resulted in significantly lower amounts of those products. Such anti-glyco-oxidative properties were also shown by meloxicam as well (↓39%, ↓17%, ↓55%, respectively). Furthermore, increased tryptophan concentration in all investigated samples versus control were observed (Figure 5D–G).

AOPP and PC contents were statistically higher in presence of BSA + glyoxal compared to control. All reviewed agents caused a significant decrease of aforementioned parameters (meloxicam: ↓55%, ↓15%). The addition of glyoxal to BSA led to reduction in total thiols concentration. Decreased levels of total thiols were noticed after inhibitors were added to BSA + GO (Figure 5H–J)

Significantly greater DPPH, TAC and FRAP were observed in comparison to BSA + GO when BSA + GO were incubated with meloxicam (↑8%, ↑13%, ↑15%), aminoguanidine (↑33%, ↑33%, ↑24%), trolox (↑28%, ↑8%, ↓15%) or reduced glutathione (↑46%, ↑53%, ↑7%, Figure 5K–M).

### 2.5. The Effects of Meloxicam and Other Additives on Protein Glycation, Glycooxidation and Oxidative Damage as Well as Total Antioxidant Potential in Methylglyoxal (MGO)-Induced Albumin Glycation

**Figure 6:**
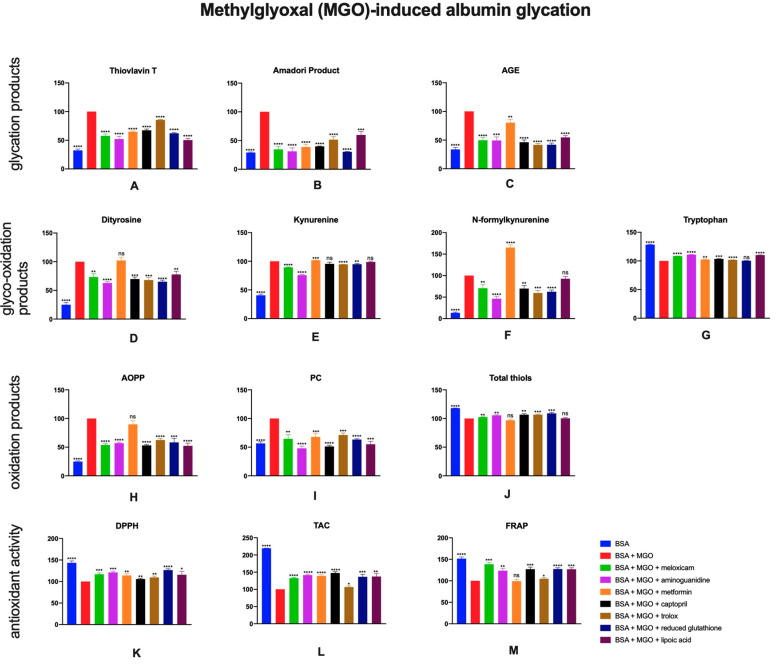
The effects of meloxicam, captopril, trolox, reduced glutathione, lipoic acid, aminoguanidine or metformin addition to BSA + methylglyoxal solution on protein glycation (**A**–**C**), glycooxidation (**D**–**G**) and oxidative damage (**H**–**J**) as well as total antioxidant potential (**K**–**M**). AGE: advanced glycation end products; AOPP: advanced oxidation protein products; BSA: bovine serum albumin; DPPH: 2,2-di-phenyl-1-picrylhydrazyl radical scavenging capacity; FRAP: ferric reducing antioxidant power; MGO: methylglyoxal; PC: protein carbonyls; TAC: total antioxidant capacity; ns: not significant vs. control; * *p* < 0.05 vs. control; ** *p* < 0.01 vs. control; *** *p* < 0.001 vs. control; **** *p* < 0.0001 vs. control.

The addition of methylglyoxal was responsible for the increased glycation markers concentrations. The study demonstrated significantly lower amounts of thioflavin T, Amadori products and AGE in samples with BSA + MGO and investigated inhibitors (meloxicam: ↓42%, ↓65%, ↓50%, respectively, Figure 6A–C).

MGO-induced albumin glyco-oxidation resulted in higher levels of dityrosine, kynurenine and N-formylkynurenine. Significantly lover contents of investigating products were observed in samples containing antioxidative substances—aminoguanidine (↓37%, ↓24%, ↓54%), captopril (↓30%, ↓5%, ↓30%) and trolox (↓32%, ↓5%, ↓40%). Similar properties were also presented by meloxicam (↓27%, ↓11%, ↓29%). Moreover, higher levels of tryptophan in BSA + MGO + meloxicam, captopril, trolox, lipoic acid, aminoguanidine or metformin were noticed (Figure 6D–G).

The presence of methylglyoxal in BSA sample was the cause of higher AOPP and PC levels. On the other hand, oxidation inhibitors, along with meloxicam (↓46%, ↓36%), were responsible for the decrease of given markers. The contents of total thiols were significantly higher in BSA and BSA + MGO + meloxicam (↑2%), aminoguanidine (↑5%), captopril (↑7%), trolox (↑7%) and reduced glutathione (↑9%) compared to BSA + MGO control sample (Figure 6H–J)

When BSA + MGO was incubated with meloxicam higher DPPH, TAC and FRAP were observed (↑17%, ↑33%, ↑38%). There were also significantly higher values of the aforementioned parameters in BSA + MGO with aminoguanidine (↑21%, ↑41%, ↑23), metformin (↑14%, ↑39%, ↓1%), captopril (↑6%, ↑48%, ↑27%), trolox (↑10%, ↑7%, ↑5%), reduced glutathione (↑27%, ↑37%, ↑27%) or lipoic acid (↑16%, ↑37%, ↑27%), excepting sample with metformin in FRAP measurement (Figure 6K–M).

### 2.6. The Effects of Meloxicam and Other Additives on Protein Glycation, Glycooxidation and Oxidative Damage as Well as Total Antioxidant Potential in Chloramine T-Induced Albumin Oxidation

**Figure 7:**
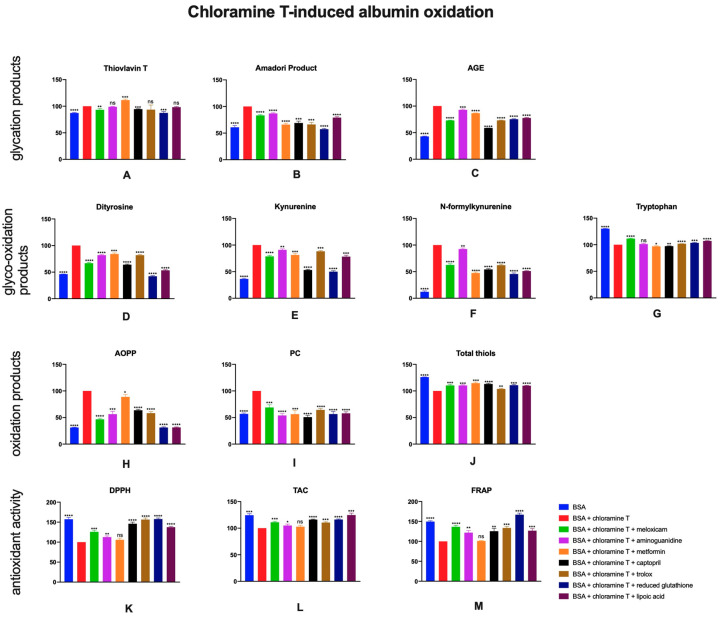
The effects of meloxicam, captopril, trolox, reduced glutathione, lipoic acid, aminoguanidine or metformin addition to BSA + chloramine T solution on protein glycation (**A**–**C**), glycooxidation (**D**–**G**) and oxidative damage (**H**–**J**) as well as total antioxidant potential (**K**–**M**). AGE: advanced glycation end products; AOPP: advanced oxidation protein products; BSA: bovine serum albumin; DPPH: 2,2-di-phenyl-1-picrylhydrazyl radical scavenging capacity; FRAP: ferric reducing antioxidant power; PC: protein carbonyls; TAC: total antioxidant capacity; ns: not significant vs. control; * *p* < 0.05 vs. control; ** *p* < 0.01 vs. control; *** *p* < 0.001 vs. control; **** *p* < 0.0001 vs. control.

Assays evaluating protein glyco-oxidation products revealed that chloramine T induces oxidation in BSA samples. Presence of meloxicam (↓26%), captopril (↓38%), trolox (↓26%), reduced glutathione (↓24%), lipoic acid (↓21%), aminoguanidine (↓9%) or metformin (↓14%) resulted in lower intensities of AGE-specific fluorescence in BSA + chloramine T solution versus control samples. Decreased concentrations of Amadori products and thioflavin T in all investigated samples (meloxicam: ↓17%, ↓7%) comparing to control sample were observed (Figure 7A–C).

The results of protein glyco-oxidation products measurement showed that amounts of dityrosine, kynurenine and N-formylkynurenine in BSA + chloramine T were significantly higher compared to BSA alone. Captopril (↓36%, ↓47%, ↓46%), trolox (↓18%, ↓12%, ↓38%), reduced glutathione (↓58%, ↓50%, ↓54%), lipoic acid (↓47%, ↓22%, ↓49%), aminoguanidine (↓18%, ↓9%, ↓7%), metformin (↓16%, ↓19%, ↓53%) or meloxicam (↓33%, ↓21%, ↓37%) caused a decrease of concentrations of glyco-oxidation products when added to BSA + chloramine T (Figure 7D–G).

Chloramine T induced a marked oxidative damage and higher AOPP and PC levels in BSA samples. Significantly lower concentrations of discussed markers were observed after inhibitors were added (meloxicam: ↓53%, ↓31%). In contrast, the total thiols level was lower in BSA + chloramine T compared to BSA. After addition of inhibitors and meloxicam (↑10%) to BSA + chloramine T, an increase of total thiols was noticed (Figure 7H–J).

Chloramine T was also the cause of decreasing DPPH, TAC and FRAP in BSA + chloramine T versus BSA. Higher levels of DPPH, TAC and FRAP were present in samples meloxicam (↑26%, ↑11%, ↑37%), aminoguanidine (↑13%, ↑5%, ↑22%), captopril (↑46%, ↑16%, ↑26%), trolox (↑57%, ↑11%, ↑34%), reduced glutathione (↑58%, ↑16%, ↑67%) or lipoic acid (↑37%, ↑25%, ↑27%) was added to BSA + chloramine T. No significant differences in total thiols, DPPH, TAC and FRAP between control and BSA + chloramine T with metformin were noticed (Figure 7K–M).

### 2.7. Validation of Results by the ELISA Method

To check the validity of results based on the fluorometric assays of BSA glyco-oxidation, the content of AGE was also determined using the ELISA method [9]. In general, the results obtained are consistent with those analyzed by the fluorimetry (Supplementary Materials, Figure S1).

## 3. Discussion

This is the first study in which the anti-glycating properties of meloxicam were assessed. We have shown that the drug strengthens the antioxidant barrier and in vitro inhibits the protein glyco-oxidation comparable to well-known antioxidants and anti-glycation agents. The antioxidant and anti-glycation activity may explain the additional properties of meloxicam.

Meloxicam is a non-steroidal anti-inflammatory drug from the oxicam group (derivatives of enolic acid). The chemical structure of meloxicam is similar to that of piroxicam, but it is a newer drug and has a better safety profile [20,30]. Although the drug inhibits the synthesis of inflammatory mediators, the exact mechanism of meloxicam is still unknown. Meloxicam has been shown to have a preferential effect to COX-2, which favors the selective inhibition of prostaglandins in tissues affected by inflammation. However, the drug may also exhibit an antioxidant activity. Hassan et al. showed that meloxicam ameliorated doxorubicin-induced renal injury via increasing antioxidant barrier in mice [22]. Another study demonstrated that meloxicam decreased aluminum- and tetrachloride-induced oxidative stress associated with rat hepatotoxicity [31,32]. Goverdhan et al. demonstrated neuroprotective effects of meloxicam through augmenting antioxidant enzymes in mice brain [33]. Nevertheless, there are no studies assessing the anti-glycation properties of meloxicam. Taking into account the increasing incidence of metabolic diseases (especially obesity, insulin resistance and diabetes mellitus) as well as the prolongation of human life, we have assessed the effect of meloxicam on protein glyco-oxidation as well as antioxidant activity. It is well known that in living organisms, the oxidation and glycation of proteins is caused by various factors (primarily sugars and aldehydes), but their mechanisms of action are often different [10]. Therefore, on the basis of a literature analysis, we have selected the most frequently used glycating (glucose, fructose, ribose, glyoxal and methylglyoxal) and oxidizing agents (chloramine T) [8,9,11,12,25,26,27,28]. Furthermore, it should be remembered that protein glyco-oxidation is a very slow process (run in the body for weeks and months) and it is difficult to study it in vitro. In our experiment, we applied a widely used and validated model [8,9,11,12,25,26,27,28] which allows us to assess the anti-glyco-oxidant properties in a short time. In addition, several biomarkers have been used to measure the glycation/oxidation rate. Indeed, the assessment of a single redox biomarker has limited and questionable value in confirming oxidative damage to proteins [34].

Albumin is the most important blood protein (65% of all plasma proteins) that regulates the colloid-oncotic pressure and blood pH. Moreover, it participates in the transport of many endogenous and exogenous substances (e.g., hormones, fatty acids and drugs) as well as shows strong antioxidant properties through its ability to bind transition metal ions [35,36]. Bovine serum albumin is the most commonly used protein in in vitro studies because of its high homology to human albumin, its high purity and the stability of prosthetic groups [37]. BSA has 35 thiol groups in its structure and 34 of these are connected via disulphide bridges. However, it is a single free thiol group of albumin, which participates in the oxidation and the connection of many ligands (e.g., drugs or pro-oxidant metals) [10,37]. In the BSA structure, tryptophan also plays an important role. Attaching the ligand molecule to the binding site causes quenching of tryptophan fluorescence and is equivalent to the ligand binding capacity of albumin [37]. Importantly, albumin is a protein that is easily oxidized and glycated in vivo. This is due to its high plasma concentration, relatively long half-life and high content of cysteine, lysine and arginine, which are particularly susceptible to oxidizing and glycating agents [10,38]. Generally, the protein glycation can be divided into several stages. The early include the formation of the Schiff base and the Amadori products, which are the first Maillard reaction products. Then, the advanced glycation end products (AGEs) are formed including furyl-furanyl-imidazole (FFI), carboxymethyl lysine (CML), pyraline and pentosidine [9,37]. However, it should be recalled that with the glycation process, the oxidation of proteins occurs simultaneously. AOPPs are the final product of this complex process, which are derivatives of oxidized albumin and arise from the accumulation of oxidized residues of dityrosine, cysteine, arginine and tryptophan [39,40].

We have shown that meloxicam inhibits protein glycation (↓amyloid-β, ↓Amadori products and ↓AGE) and glyco-oxidation (↓dityrosine, ↓kynurenine, ↓N-formylkynurenine and ↑tryptophan) and as well as reduces the content of protein oxidation products (↓AOPP, ↓PC and ↑thiol groups) when exposed to all glycating and pro-oxidant factors. Meloxicam also increases the antioxidant properties of albumin (↑DPPH, ↑TAC, ↑FRAP). Interestingly, the antioxidant and anti-glycation activity is similar to that of routinely used antioxidants.

The main glycating sugar in the human body is D-glucose. Since only a small amount of D-glucose is present in a free aldehyde form, in living organisms, glycation occurs very slowly over many weeks. Nevertheless, in conditions with increased blood glucose levels, the glycation of proteins is increased [41]. Interestingly, enhanced glycation/oxidation of proteins was observed not only in patients with metabolic diseases, but also autoimmune, neurodegenerative, rheumatic and inflammatory disorders [19,42,43,44,45]. However, fructose and ribose are much more reactive than glucose, as confirmed by the results of our study. Indeed, the highest intensity of glycation processes was observed in BSA incubated with ribose, the lowest with fructose and the least with glucose. Such observations are consistent with those of other authors [8,27,41]. Moreover, this is reflected in the prevention of protein glycation in vitro. As expected, inhibition of glyco-oxidation by meloxicam was significantly lower in ribose-induced albumin glycation compared to fructose or glucose models. Similar changes have also been observed for the other additives.

Not only sugars, but also various bicarbonyl derivatives formed during glucose autoxidation are responsible for protein glycation processes. These include primarily oxoaldehyde (glyoxal) and its methylated derivative (methylglyoxal), which are direct precursors to the formation of Amadori products and advanced glycation end products [13,46]. Interestingly, glyoxal and methylglyoxal are also substrates for many oxidoreductases; thus, in vivo, they can also induce disturbances in cellular redox homeostasis [47]. We have shown that meloxicam reduces carbonyl stress in BSA samples incubated with glyoxal and methylglyoxal solutions. Indeed, the fluorescence of glyco-oxidatively modified amino acids as well as the content of protein oxidation products were significantly lower after meloxicam treatment. We also observed an increase in the tryptophan fluorescence, which indicates conformational changes in glyco-oxidized albumin and a reduced glycation of this protein. Importantly, the anti-glycating properties of meloxicam were comparable to those of aminoguanidine. Although this compound very effectively blocks the formation of AGEs (reacting with ketones and aldehydes), it is not used in clinical practice due to its very high cytotoxicity [48]. Meloxicam has a much better safety profile with similar anti-glycation properties. The main side effects of meloxicam are gastrointestinal complaints and hypersensitivity reactions. Although the drug is generally well tolerated, meloxicam must be safely used in patients allergic to NSAIDs, in patients prone to gastrointestinal bleeding/peptic ulcer disease as well as in patients with severe liver and kidney failure [20,30]. Although our research cannot explain this accurately, the increased antioxidant properties of meloxicam may result from the ring-shaped structure of the molecule having conjugated double bonds, as well as from the presence of functional groups in these rings (Figure 1).

Advanced oxidation and glycation protein products show the ability to aggregate and accumulate, impairing the functioning of many human organs. Due to structural similarity, both AGEs and AOPPs combine with a specific receptor (RAGE) to stimulate the proinflammatory NFkB signaling pathway [13,49,50]. Its activation not only increases the production of cytokines and chemokines, but also results in increased secretion of prothrombotic factors and oxygen free radicals [13,50,51]. Interestingly, protein glyco-oxidation is not only intensified under metabolic disturbances (e.g., oxidative stress, hyperglycemia, insulin resistance, diabetes mellitus or abnormal lipid metabolism). Indeed, bicarbonyl derivatives (GO, MGO) as well as AGEs may also be of exogenous origin. They can be derived from food products such as candies, pastries, fizzy drinks, processed meat or fast foods [52]. Thus, there is a great interest in compounds with anti-glycating and antioxidant properties. Since meloxicam inhibits both protein glycation and oxidation, it can be considered for patients with metabolic diseases who need an anti-inflammatory and analgesic drug. Nevertheless, this issue requires further research including possible side effects and drug interactions.

Our study also confirms previous reports of the ability of metformin to inhibit protein glyco-oxidation. Although this compound reduces protein glycation in vivo, it does not show this effect in vitro.

Since fluorometric measurements of BSA glyco-oxidation may result from interference by additives [9], the content of AGE was also determined using the ELISA method. Generally, the results obtained are consistent with those analyzed by the fluorometric determination. This is also confirmed by the results of other studies [8,9,27].

All NSAIDs, including meloxicam, have an anti-inflammatory effect by inhibiting the activity of cyclooxygenases and thus reducing prostaglandin production [20]. However, it should be remembered that blocking prostaglandin synthesis is not able to completely inhibit the inflammatory process. Indeed, an inflammatory reaction involves many phases in which migration, adhesion, diapedesis and chemotaxis of cells occur, as well as a humoral response in which histamine, complement proteins, cytokines, prostacyclin, prostaglandin, biogenic amines as well as free radicals are released [53]. However, free radicals triggered by neutrophils and macrophages play a key role in inflammatory reactions. Indeed, ROS enhance the inflammatory response by increasing the production of cytokines and chemokines (in the positive feedback mechanism) and also damage local cells through oxidation and nitration [53,54]. Given that inflammation has a complex pathogenesis, pleiotropic activity of meloxicam may increase the effectiveness and efficiency of anti-inflammatory treatment.

Meloxicam is typically used for the symptomatic treatment of rheumatoid arthritis and ankylosing spondylitis [20]. Pharmacokinetic studies have shown that meloxicam binds strongly to plasma proteins and also penetrates into the synovial fluid, reaching a concentration of about half the plasma level [55,56]. Meloxicam is also characterized by a relatively long half-life (about 20 h), which explains its long therapeutic effect [20,30]. Given the key contribution of oxidative stress/protein glycation in the pathogenesis of rheumatic diseases [57,58], meloxicam can act not only by reducing inflammation, but also by antioxidant action.

In conclusion, we have shown that meloxicam enhances the antioxidant properties of albumin and prevents the protein oxidation and glycation under the influence of various factors such as sugars, aldehydes and oxidants. In general, the effect of meloxicam is comparable to that of other anti-glycating agents or free radical scavengers. The next step of research is to conduct in vivo studies assessing the anti-glycating and antioxidant properties of meloxicam in animal and human models and the establishment of the dose–activity relationship of meloxicam. Indeed, it should be noted that the glycation agents used in the study are in much higher concentrations than physiological ones [8,9,27]. Although this experimental model is commonly used to assess the anti-glyco-oxidant properties of additives [8,9,11,12,25,26,27,28], it will never replace research on living organisms. Nevertheless, taking into account the strong anti-glycation activity of meloxicam in vitro, modifications of the molecule structure can also be considered to diminish the anti-inflammatory effect (in favor of an anti-glycating action) and thus reduce the adverse effects of the drug. The use of meloxicam in new therapeutic indications still remains an open question.

## 4. Methods

### 4.1. Reagents and Equipment

All chemicals were of analytical grade and purchased from Sigma-Aldrich (Nümbrecht, Germany/Saint Louis, MO, USA). Immediately before use, solutions were sterilized by filtration through 0.2 mm membrane filters (Biosens). The absorbance/fluorescence was measured using Infinite M200 PRO multimode microplate reader (Tecan Group Ltd., Männedorf, Switzerland).

### 4.2. Bovine Serum Albumin (BSA)

The glycated/oxidized BSA formation was performed according to a previously published method [8,9,27,28,29,59]. Briefly, BSA (purity of 96%) was dissolved in sodium phosphate buffer (0.1 M, pH 7.4) containing 0.02% sodium azide (as a preservative). Glucose (Glc), fructose (Fru), and ribose (Rib), as well as glyoxal (GO) and methylglyoxal (MGO), were used as glycating agents. For measurements of the effects of additive on the process of protein glycation, BSA was incubated with 1 mM meloxicam and 0.5 M sugars (Glc, Fru, Rib) for six days or 2.5 mM GO and MGO for 12 h [8,9,12]. GO and MGO were used within a month after delivery and their working solutions were prepared immediately before assays [27]. For measurements of the effects of additive on protein oxidation, BSA and meloxicam was incubated with 20 mM chloramine T for 60 min [11]. All samples were incubated darkly in the closed vials at a temperature of 37 °C with continuous shaking (50 rpm) [8,9,27,28,29]. The incubation mixtures contained BSA at a final concentration of 0.09 mM. Concentrations of glycating agents as well as the optimal incubation conditions for studies of the modification of the glycoxidation rate by additives were determined and validated based on the previously published kinetic studies [8,27]. Although the concentrations of sugars, aldehydes and oxidants are much higher than physiological levels, they are convenient for modelling in a relatively short time the physiological processes occurring in the human body over weeks or months [8,9,27]. Such experimental conditions are routinely used to determine the anti-glyco-oxidant properties of new substances [8,9,11,12,25,26,27,28]. To compare the results obtained for meloxicam, aminoguanidine and metformin were used as a known inhibitor of protein glycation, while captopril, Trolox, reduced glutathione and lipoic acid as an antioxidant [8,9,27,28,29,59]. The concentration of all additives was 1 mM and established on the basis of the other in vitro studies, in proportion to the high concentrations of the glycating agents [8,9,11,12,25,26,27,28]. The experiment was performed in three series, each time in duplicate.

### 4.3. Protein Glycation Products

#### 4.3.1. Thioflavin T (Amyloid-β Formation)

Thioflavin T assay was performed in order to measure fluorescence emitted when amyloid fibrils or oligomers are bound to thioflavin T. 90 μL of investigated samples and 10 μL of Thioflavin T were mixed and placed in a microplate. Fluorescence intensity was measured at 385/485 nm [60,61].

#### 4.3.2. Amadori Products

Total content of Amadori products was estimated using colorimetric method with Nitro Blue Tetrazolium (NBT) assay. Characteristic absorbance was measured at 525 nm with the use of monoformazan extinction coefficient (12,640 M^−1^cm^−1^) [46].

#### 4.3.3. Advanced Glycation End Products (AGE)

Advanced glycation end products (AGE) content was analyzed spectrofluorometrically. AGE-specific fluorescence was measured at 440/370 nm in a 96-well microplate reader [62,63]. Prior to the reading, samples were diluted with PBS (1:5, *v:v*) [64]. AGE content was also assayed using the ELISA method (USCN, Life Science, Wuhan, China), according to the manufacturer’s instructions.

### 4.4. Protein Glyco-Oxidation Products

#### Kynurenine, N-Formylkynurenine, Dityrosine and Tryptophan

Kynurenine, N-formylkynurenine, dityrosine and tryptophan were detected by measuring the fluorescence emission and excitation at 365/480, 325/434, 330/415, 95/340 nm, respectively. Prior to the measurement, samples were diluted with 0.1 M sulphuric acid (1:5, *v:v*). Results were standardized to fluorescence of 0.1 mg/mL quinine sulphate in 0.1 M sulphuric acid [65].

### 4.5. Protein Oxidation Products

#### 4.5.1. Advanced Oxidation Protein Products (AOPP)

In order to examine the concentration of AOPP, a spectrophotometric detection was performed. 200 μL of the investigated solutions diluted with PBS in 1:5 ratio (*v:v*), chloramine-T standard solutions (0–100 μmol/L) and 200 μL of blank PBS were transferred into the 96-well microplate. Then, 10 μL of 1.16 M potassium iodide as well as 20 μL of acetic acid were added to the wells. The absorbance was read immediately in a microplate reader at 340 nm wavelength in relation to a blank (200 μL PBS, 10 μL potassium iodide, 20 μL acetic acid). Chloramine-T solutions presented linear absorbance in the range of 0–100 μmol/L [62].

#### 4.5.2. Protein Carbonyls (PC)

Determination of PC was performed using the reaction of carbonyls with 2,4-dinitrophenylhydrazine (2,4-DNPH) in proteins that underwent oxidative damage. The absorbance of the reaction products was measured colorimetrically at 355 nm with absorption coefficient for 2,4-DNPH (22,000 M^−1^cm^−1^) [66].

#### 4.5.3. Total Thiols

Total thiols were evaluated colorimetrically at 412 nm wavelength with the use of Ellman’s reagent in 0.1 M phosphate buffer. The content of thiol groups was determined based on a standard curve for reduced glutathione (GSH) [67].

### 4.6. Total Antioxidant Potential

#### 4.6.1. 2,2-di-phenyl-1-picrylhydrazyl (DPPH) Radical Scavenging Capacity

Determination of free radicals scavenging activity was performed based on Brand-Williams method. 390 μL methanolic dilution of DPPH and 10 μL of each sample were mixed and transferred to 96-wells microplate. The reaction mixture was kept in the dark at room temperature for 30 min. The absorbance was measured at 515 nm [68,69].

#### 4.6.2. Total Antioxidant Capacity (TAC)

TAC of each sample was determined following Erel method. 2,2-azinobis(3-ethylbenzo-thiazoline-6-sulfonate) (ABTS) radical cation decolorization assay was performed to determine TAC of each investigated sample. ABTS^+^ was obtained by reacting ABTS with potassium persulfate and left at room temperature for 12 h. ABTS^+^ stock solution diluted with phosphate-buffered saline was used as control with an absorbance of 0.70 at 735 nm. The absorbance readings were taken at 735 nm after the initial mixing of 1 mL of diluted ABTS^+^ and 10 μL of sample. Results of decolorization were linear with increasing Trolox concentrations [70,71].

#### 4.6.3. Ferric Reducing Antioxidant Power (FRAP)

The ferric reducing antioxidant power of each sample was determined according to Benzie & Strain method. In order to prepare working FRAP reagent, 25 mL acetate buffer, 2.5 mL FeCl_3_·6H_2_O solution and 2.5 mL TPTZ solution were mixed and warmed to 37 °C. 300 μL FRAP reagent and 10 μL of sample diluted with 30 μL H_2_O were transferred into 96-well microplate. Absorbance readings were taken at 593 nm wavelength. The change of absorbance was calculated for each sample and related to absorbance of Fe^II^ standard solution [72].

### 4.7. Statistical Analysis

The results were expressed as a percentage of the corresponding control values (BSA + glycating agent). Differences between groups were assessed by one-way ANOVA followed by Tukey’s post-hoc test for multiple comparisons. *p* < 0.05 was considered to be statistically significant. Multiplicity adjusted *p* value was also calculated. The analysis was conducted using the statistical package: GraphPad Prism 8.3.0 for MacOS (GraphPad Software, La Jolla, CA, USA).

## Supplementary Materials

The following are available online at https://www.mdpi.com/1424-8247/13/9/240/s1, Figure S1. Validation of results by the ELISA method.
