# Supplementary material for: A New Insight into Meloxicam: Assessment of Antioxidant and Anti-Glycating Activity in In Vitro Studies
Source: Pharmaceuticals (Basel). 2020 Sep 10;13(9):240. doi: 10.3390/ph13090240 (PMC7558080; doi:10.3390/ph13090240)
Supplement: Supplementary file 1 [file pharmaceuticals-13-00240-s001.pdf]

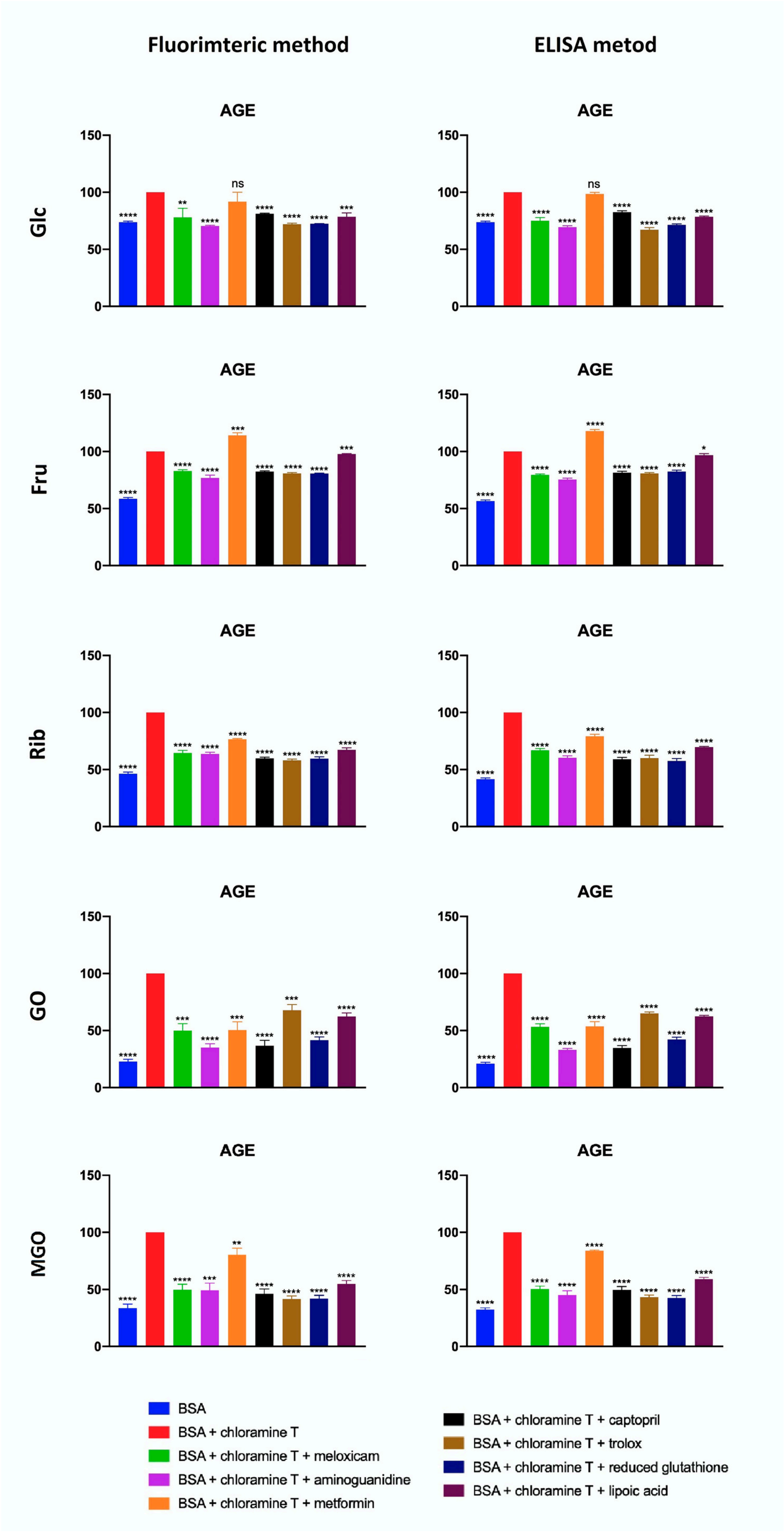
Fluorimteric method
ELISA metod
AGE
Glc
Fru
Rib
GO
MGO
0
50
100
150
ns
BSA
BSA + chloramine T
BSA + chloramine T + meloxicam
BSA + chloramine T + aminoguanidine
BSA + chloramine T + metformin
BSA + chloramine T + captopril
BSA + chloramine T + trolox
BSA + chloramine T + reduced glutathione
BSA + chloramine T + lipoic acid

Figure S1. Validation of results by the ELISA method.

The effects of meloxicam, captopril, trolox, reduced glutathione, lipoic acid, aminoguanidine or metformin addition on the content of advanced glycation end products (AGE) measured by fluorimetric method and ELISA. AGE: advanced glycation end products; BSA: bovine serum albumin; Glc: glucose-induced glycation; Fru: fructose-induced glycation; Rib: ribose-induced glycation; GO: glyoxal-induced glycation; MG: methylglyoxal-induced glycation; chloramine T: chloramine T-induced oxidation; ns: not significant vs control (BSA + glycating agent); ** $p < 0.01$ vs control; *** $p < 0.001$ vs control; **** $p < 0.0001$ vs control.
